# Re-expression of CD14 in Response to a Combined IL-10/TLR Stimulus Defines Monocyte-Derived Cells With an Immunoregulatory Phenotype

**DOI:** 10.3389/fimmu.2019.01484

**Published:** 2019-06-28

**Authors:** Sören Krakow, Marie L. Crescimone, Charlotte Bartels, Verena Wiegering, Matthias Eyrich, Paul G. Schlegel, Matthias Wölfl

**Affiliations:** Department of Hematology, Oncology and Stem Cell Transplantation, University Children's Hospital, University of Würzburg, Würzburg, Germany

**Keywords:** regulatory dendritic cells, MDSC, monocyte-derived DC, IL-10, macrophages

## Abstract

Interleukin 10 is a central regulator of the antigen-presenting function of myeloid cells. It exerts immunomodulatory effects *in vivo* and induces a regulatory phenotype in monocyte-derived cells *in vitro*. We analyzed phenotype and function of monocytic cells *in vitro* in relation to the cytokine milieu and the timing of TLR-based activation. In GM-CSF/IL-4 cultured human monocytic cells, we identified two, mutually exclusive cell populations arising from undifferentiated cells: CD83^+^ fully activated dendritic cells and CD14^+^ macrophage like cells. Re-expression of CD14 occurs primarily after a sequential trigger with a TLR signal following IL-10 preincubation. This cell population with re-expressed CD14 greatly differs in phenotype and function from the CD83^+^ cells. Detailed analysis of individual subpopulations reveals that exogenous IL-10 is critical for inducing the shift toward the CD14+ population, but does not affect individual changes in marker expression or cell function in most cases. Thus, plasticity of CD14 expression, defining a subset of immunoregulatory cells, is highly relevant for the composition of cellular products (such as DC vaccines) as it affects the function of the total product.

## Introduction

Cells of myeloid origin acquire immunostimulatory and immunoregulatory functions depending on the respective milieu. Differentiated type 1 cells, such as type 1 macrophages and dendritic cells, are essential to mount an inflammatory, and antigen-specific response (1). Alternatively activated macrophages, myeloid-derived suppressor cells (MDSCs), and regulatory dendritic cells (DCs) exert multiple immunoinhibitory functions (2–4). In human disease, these cells effectively link innate, and adaptive immunity: e.g., immunosuppressive tumor-associated macrophages can be found in various tumor-entities (5), and MDSCs circulate the blood of cancer patients (4). In contrast, alloreactivity in acute GvHD may be partially based on the dysbalance of the myeloid cell compartment after stem cell transplantation (6, 7).

Monocyte-derived cells, generated *in vitro*, share many of the characteristics of naturally occurring myeloid cell types. Once activated, monocyte-derived dendritic cells are capable of mounting a primary T-cell response, making them central to many tumor vaccination efforts. Alternative culture protocols lead to a regulatory functional profile, providing a cellular tool to address auto- and alloreactivity. As monocytes are readily available, these approaches are being evaluated in clinical trials (8).

Interleukin-10 (IL-10) is a master regulator for generating immunomodulatory cells. Depending on the culture conditions and the timing of IL-10 contact, monocyte-derived cells acquire different phenotypical and functional properties. Nomenclature is ambiguous, making it difficult to draw a general picture. Monocyte-derived macrophages are usually generated by culture with M-CSF and IL-10, whereas GM-CSF and IL-4 is thought to promote a type 1 macrophage/dendritic cell phenotype (9). Within protocols using GM-CSF/IL-4-cultured monocytic cells, the timing of the first contact with IL-10 appears to be crucial: when added directly to CD14^+^ monocytes, differentiation toward a full dendritic phenotype is thwarted. The cells are described as expressing less costimulatory molecules and less HLA-DR and maintain CD14 (10). Functionally, reduced T-cell stimulatory capacity is documented. Such monocyte-derived cells differentiated with GM-CSF, IL-13, and IL-10, have been simply classified as “macrophages” by Allavena et al. (11). Recently, using a similar approach with GM-CSF, IL-4, and IL-10 (from the start of culture) Heine et al. describe the resulting cells as CD14^+^HLA-DR^low^ “MDSC-like” cells (12). MDSC have been initially described in the murine system, whereas MDSC in humans still lack definitive classification (4). However, some subsets such as such as LIN^−^HLA-DR^−/low^, CD14^+^HLA-DR^−/low^, and CD15^+^HLA-DR^−/low^ have been defined (13). CD14^+^HLA-DR^low^ MDSC have been identified in patients with various cancer types and are associated with a less favorable prognosis (4, 14).

In somewhat parallel investigations, it was noted, that immature dendritic cells, developing under the influence of GM-CSF/IL-4, may be directed toward a regulatory phenotypic and functional profile, once they are in contact with IL-10 (15). As before, it was noted that costimulatory molecules are downregulated, while expression of inhibitory molecules such as ILT4 (16), and PD-L1 increases (17). Again, a robust immunoinhibitory capacity has been noted, as T-cell tolerance is induced. Cells generated with this type of protocol were termed “regulatory dendritic cells,” as a fraction, but by far not all, of the cells will express the DC-marker CD83.

In this study, we distinguish between different cell populations arising from standard culture conditions of human GM-CSF/IL-4 cultured monocytic cells in response to IL-10 and an activating trigger. The monocytic cells were primed with IL-10 shortly before triggering them via a TLR. Surprisingly two mutually exclusive populations with distinct phenotypic profiles can be distinguished: CD14^+^ cells matching in many aspects the phenotypical and functional aspects of MDSC/DC_reg_ and CD83^+^ cells, displaying markers of type 1 DC. This CD14^+^ cell population arises from non-differentiated cells, that had already downregulated CD14 as a consequence of GM-CSF/IL-4 culture and then re-express CD14. A fraction of these CD14^+^CD83^−^ cells can routinely be detected following certain TLR-triggers (such as R848 or LPS) even without exogenously added IL-10, but a binary signal from IL-10 and a TLR-trigger is required for maximal differentiation toward this cell type.

Using CD14 as the defining positive marker, we show that rather than a direct effect of IL-10 on individual markers or a specific function, IL-10 shifts a whole cell population toward this altered CD14^+^ phenotype, while, contrary to the paradigm, many individual markers within this population remain unaffected from the exogenous IL-10.

## Materials and Methods

### Terminology

Classification and terminology of dendritic cell and macrophage subsets remain a matter of intense discussions. Historically, monocyte-derived DCs were described either as “immature,” when treated with GM-CSF and IL-4 only, or “mature” when an activating stimulus had been provided (17). Monocyte-derived cells treated with modifying molecules such as IL-10, rapamycin or corticosteroids have been termed “regulatory DCs.” A unified nomenclature based on ontogeny has been proposed, which only refers to these cells as “monocyte-derived” (18). For the clearest terms possible, we will refer to the cells evaluated in this work as follows: all cell populations used in this work are human, monocyte-derived cells (moC). Generally, the starting population are cells cultured in GM-CSF/IL-4 containing medium (formerly ‘immature DC'), which we refer to as “_GM/IL4_moC.” Any further treatment (e.g., with IL-10 or R848) replaces the GM/IL-4 indicator (e.g., _IL10/R848_moC) implying that this treatment was added on top of the GM/IL-4 culture. If various activating conditions are summarized, “act” is put instead of the specific stimulus (e.g., _IL10/act_moC). Once cells are stimulated, we refer to them as “activated” rather than “mature.” Morphological distinctions based on CD14 and CD83 expression are added, when these subgroups are evaluated separately (e.g., _IL10/R848_moC^CD14+^). Functional differences such as a more regulatory or inflammatory profile, are discussed as functions in the paper but are not part of the terminology.

### Cell Culture

Peripheral blood mononuclear cells were obtained and cryopreserved from healthy donors, who had been eligible to donate blood in the local blood bank, by washing out leucocyte depletion filter chambers that collect leucocytes as a by-product to platelet collection. Experiments performed with such leucocytes, following pseudonymization of the donor, do not require informed consent according to a decision of our IRB. For the generation of moC, standard procedure was to allow cells to adhere to 6-well plastic dishes for 2 h and subsequently remove the non-adherent fraction by washing. Cells were then cultured in DC Medium (Cellgenix, Freiburg, Germany), supplemented with 1% human serum (Biochrom, Berlin, Germany) and 800 U/ml GM-CSF (Gentaur, Aachen, Germany) and 100 U/ml IL-4 (Peprotech, Hamburg, Germany). Forty eight hours after initiation of the culture, fresh medium was added, including GM-CSF and IL-4. For _IL10/R848_moC, IL-10 (40 ng/ml; Peprotech, Hamburg, Germany) was added at least 1 h before adding the activation stimulus. As activation stimuli, the following reagents were used: R848 (2 μg/ml; Invivogen, San Diego, CA, USA); LPS(*E. coli*) (30 ng/ml; Sigma, St. Louis, Missouri, USA); MPLA-SM (1 μg/ml, Invivogen, France); Poly(I:C) (HMW, 10 μg/ml, Invivogen, France). Additional cytokines used in the assays were: TNFα (10 ng/ml; Peprotech, Hamburg, Germany), IL-1ß (10 ng/ml, Cellgenix, Freiburg, Germany). Cells were evaluated 16–48 h after activation, depending on the individual question of the assay. Functional grade anti-IL10-antibody and anti-IL10R-antibody was purchased from eBioscience.

### Flow Cytometry

Analysis of cell cultures was performed on a FACS Canto II flow cytometer (BD) using 3 lasers. Staining protocols followed standardized procedures at optimized antibody concentrations. The antibodies against the following antigens were used: CD14 (PE; MϕP9; BD Biosciences), CD36 (PerCpCy5.5; eBioNL07; eBioscience), CD80 (PerCpCy5.5; 2D10; Biolegend) CD83 (Brilliant Violet 421TM; HB15e; Biolegend) CD85d (APC; 42D1; eBioscience), CD85k (APC; ZM4.1; eBioscience) CD86 (PerCPCy5.5; IT2.2; Biolegend), CD91 (APC; A2MR-a2; eBioscience), CD163 (FITC; GHI/61; Biolegend), CD206 (FITC; 15-2; Biolegend), CD273 (APC; MIH18; Biolegend) CD274 (FITC; MIH1; BD Biosciences), CD279 (FITC; MIH4; eBioscience), CX3CR1 (PerCpCy5.5; 2A9-1; Biolegend) Viability Dye (eFluor 780; eBioscience).

### Endocytosis Assay

Experimental groups were seeded in 96 well-plates using DC medium without serum or cytokines. APC-Dextran (MW: 10,000; 200 μg/ml; Invitrogen) was added at t0. At defined time points (0, 20, 40, 60, 90 min), cells were harvested and immediately washed using cold PBS and placed on ice until FACS analysis.

### ELISA

For IL-6 ELISA, supernatant from the differentially activated groups (3 × 10^6^ cells/group) was frozen and later analyzed. ELISAs were performed using kits from ThermoFisher, following the manufacturer's protocol.

### T-Cell-Assays

Priming of naïve T-cells was performed following the protocol published previously in detail (19, 20). Briefly, CD45RO^−^CD57^−^CD8^+^ T-cells were stimulated at a 10:1 ratio with moC, pulsed with the HLA-A2-restricted, heteroclitic peptide Melan-A_(26−35(A27L)_, immunograde (ELAGIGILTV; jpt, Berlin, Germany). Cells were grown in Cellgenix GMP DC Medium (Cellgenix, Freiburg, Germany). IL-21 (Peprotech, Hamburg, Germany) was added at the start of culture. IL-7 and IL-15 (both Peprotech, Hamburg, Germany) was added on day 3 of culture and refreshed every 2–3 days. Cells were analyzed on day 10 of culture, taking cell counts and performing MHC-multimer-staining (Immudex, Copenhagen, Denmark).

### Statistics

Statistical analysis was performed using GraphPad Prism. Error bars always indicate standard deviation. *T*-test or two-way ANOVA was chosen as appropriate and analysis was done taking paired observations into account and correcting for multiple comparisons (Tukey).

## Results

### CD14 Is Re-expressed on moC Following an IL10/R848 Trigger

**Figure 1:**
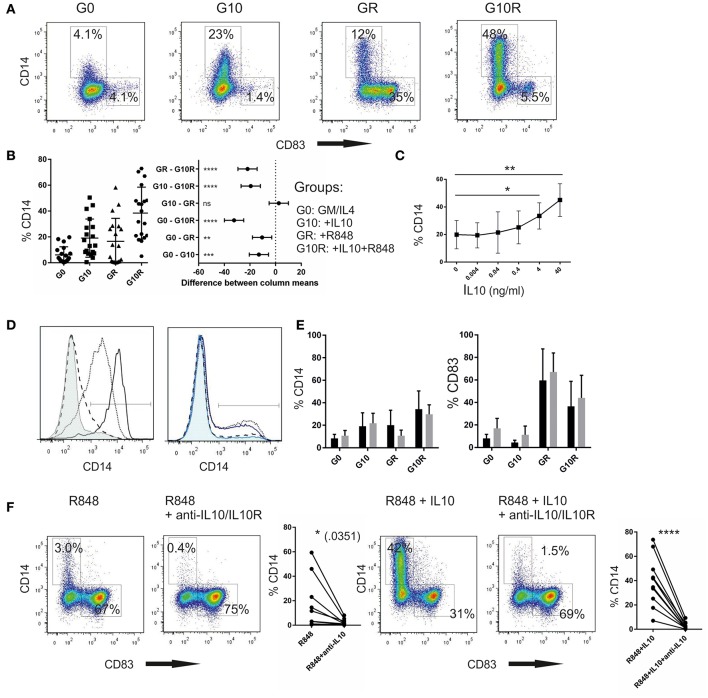
IL-10 in combination with R848 induces re-expression of CD14 in GM-CSF/IL4-cultured monocytic cells **(A)**. Individual plots of cells on d5 of culture after 24 h-incubation R848 (2 μg/ml) without and with IL-10 (40 ng/ml) pre-incubation (1 h), or the combination **(B)**. Summary of 19 different experiments from different healthy donors. (Two-way ANOVA for multiple comparisons; *p < 0.05; **p < 0.01; ***p < 0.001; ****p < 0.0001) **(C)**. IL-10 dose dependent increase of the percentage of CD14^+^ cells in combination with a fixed dose of R848 (2 μg/ml) **(D)**. Left: Downregulation of CD14 on monocytes during culture in GM-CSF/IL-4 (before experimental treatment): %CD14^+^: black solid: d1 (94%); dotted: d2 (71%); dashed: d3 (12%); thin solid, tinted: d5 (without activation) (8.6%) (one of 3 experiments). Right: Upregulation of CD14 on day 5 of culture in cells, after treatment on day 4: dotted: IL-10/R848 (33%); solid blue: IL-10/R848 treated, after CD14 depletion on d4 (27%); dashed: R848 only (15%), light blue,tinted: R848(only) after CD14-depletion on d4 (10%) **(E)**. Comparison of %CD14^+^ cells (left) and %CD83^+^ cells (right) after the respective treatment following a 4 day (black) culture or a 7 day (gray) culture in GM/IL-4 (*n* = 3) **(F)**. Effect of IL-10 blockade on CD14 re-expression. Functional grade anti-IL10-antibody and anti-IL10R-antibody were added prior to preincubation with IL-10 or prior to R848 addition. CD14 and CD83 expression were measured 16 h later. Examplary plots and a summary from 7 different donors are shown.

For all experiments shown here, human monocyte-derived dendritic cells were generated by selecting the plastic adherent fraction of the cells as described in the methods. The resulting adherent cell population predominantly expresses CD14, which is gradually lost throughout the culture in GM-CSF and IL-4 containing medium. Sometimes retained CD14 expression is reported (8, 10) and is attributed to incomplete differentiation due to culture conditions. Therefore, we initially thought of CD14 as a marker that is gradually lost when monocytes differentiate toward dendritic cells and we expected to see a differentiation stop once IL10 is added to the culture. Indeed, when IL-10 alone was added on day 3 for 24 h, we noticed a higher fraction of CD14^+^ cells, as was already outlined in earlier work (15). However, we also noticed a fraction of the moC expressing even higher levels of CD14, once they had been stimulated with the TLR7/8 agonist R848 and this fraction significantly increased when the cells were pre-incubated with IL-10 followed by R848, an example of which is shown in Figure 1A. CD14 expression was mutually exclusive to CD83 expression, as a marker for fully activated DC. To assess whether these differences were truly dependent of the culture conditions, or whether factors inherent to different donors contributed to the results, we repeated this experiment with cell preparations from 19 different donors. Experiments were performed by 3 different researchers. As shown in Figure 1B, the range of CD14 expression for each individual donor is high in each of the experimental groups. Specifically _GM/IL4_moC, without any additional manipulation, showed a mean CD14 expression of 4.6% with a standard deviation of 5.5. One explanation may be, as discussed later, that donor-inherent factors (e.g., current *in vivo* cytokine milieu at the time of donation) may influence cell differentiation *in vitro*. Despite this rather large inter-donor variation, the effect of IL-10 on upregulation of CD14, especially when combined with R848 activation, was highly statistically significant (*p* < 0.0001, Two-way ANOVA; Figure 1B, right panel). Re-expression of CD14 was dose-dependent, with most robust effects starting in the range of 4–40 ng/ml of IL-10 (Figure 1C). These CD14^+^ cells emerge from the CD14^−^ population, as during culture in GM-CSF/IL-4 CD14-expression is rapidly lost (Figure 1D, left). Even if residual CD14^+^ cells are depleted, using CD14-microbeads prior to IL-10 exposure (day 3), re-expression of CD14 occurs within 24 h after incubation with IL-10 and R848 (Figure 1D, right). Nevertheless, one might argue that 4-day cultured cells are still too undifferentiated and the observed results may be partially affected by incomplete downregulation. We, therefore, prolonged cell culture with GM-CSF and IL-4 for 7 days, and then reevaluated CD14 expression in relation to IL-10 and/or R848. Seven-day-cultured _GM/IL4_moC expressed even less CD14 and adding either IL-10 or R848 alone only resulted in a slight increase in CD14^+^ cells. Combining IL-10 and R848, we observed a similar increase in CD14^+^ cells after a 7-day culture period (Figure 1E) to what we had observed in multiple donors in 4-day cultured cells (Figure 1B). Likewise, CD83 upregulation occurred independently of the culture time (4 vs. 7d) but was hindered by IL-10, as has been described in many papers. Of note, excess amounts of GM-CSF or IL-4 (10-fold) had no effect; specifically, it did not counteract the observed upregulation of CD14 (three experiments, data not shown).

As we observed a small percentage of CD14^+^ cells following activation with R848 only, we suspected that this fraction responded to endogenous IL-10 produced upon TLR-triggering. Experimentally this was confirmed by blocking IL-10 signaling using anti-IL-10- and anti-IL-10R-antibodies. Original plots of one representative experiment, as well as the summary of all 7 experiments are shown in Figure 1F. Even with the rather big variation of the CD14^+^ fraction following R848 activation, the results suggest a significant effect of IL-10 blockade in conditions were no exogenous IL10 was added (left panels). As controls, we also show the experiments with exogenous IL10 added, and then blocked, which was highly statistically significant. We conclude that endogenous IL10, produced during stimulation with R848, contributes to upregulation of CD14 in a fraction of these cells.

### CD14 Re-expression Depends on the Activating Signal and the Pre-existing Cytokine Milieu

**Figure 2:**
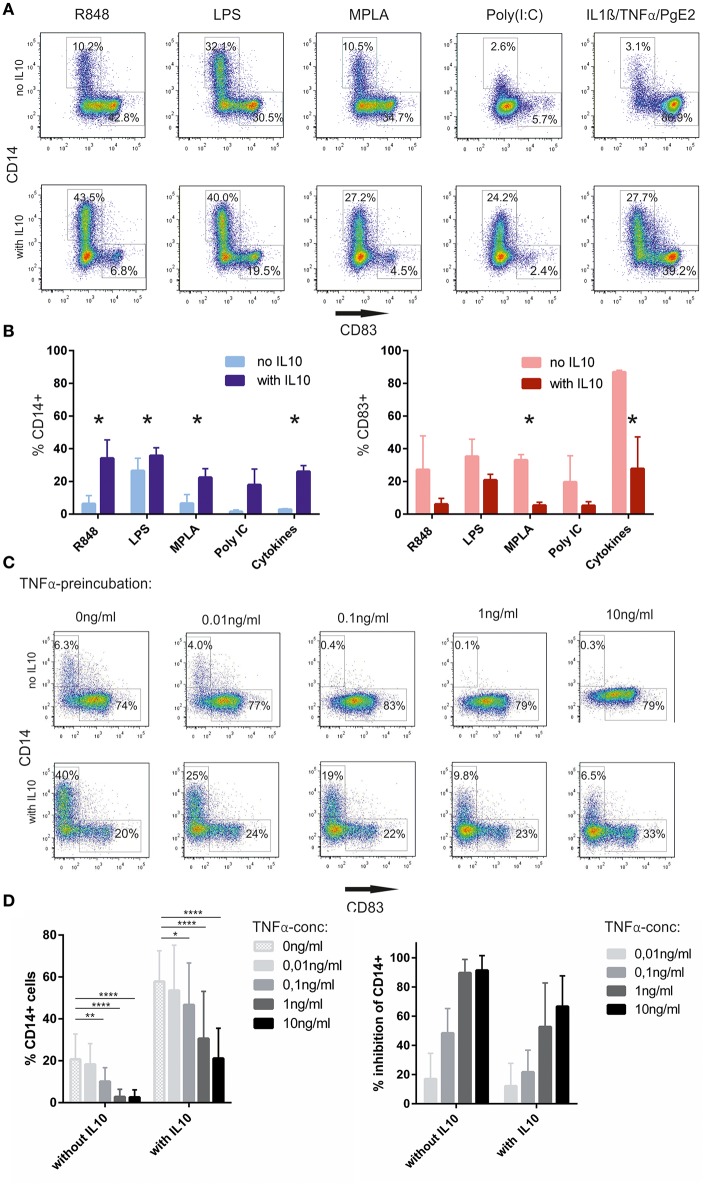
CD14 upregulation depends on IL-10 and the maturation stimulus **(A)**. Monocytes, cultured in GM-CSF/IL-4, were either preincubated with IL-10 or not, and subsequently stimulated for 16–18 h with the indicated substances. Cells were evaluated for CD14 and CD83 expression the following day **(B)**. Summary from *n* = 3 experiments **(C)**. TNFα-preincubation for 24 h prior to adding IL10 hinders CD14 upregulation. Upper row: no IL10 addition, lower row with IL-10 **(D)**. Summary of 5 independent experiments, showing the absolute % of CD14 depending on TNFα-preincubation without or with exogenous IL-10 (left, Two-way-ANOVA for multiple comparisons, *p < 0.05; **p < 0.01; ***p < 0.001; ****p < 0.0001). The right panel shows the relative inhibition of CD14 expression by TNFα, taking the %CD14 without TNFα of each individual experiment as the reference point (100%). % Inhibition is calculated as: [1-(%CD14(sample)/%CD14(ref.point)] × 100. As these are calculated values from the original data shown in the left panel, no statistical test is shown in this panel.

We next asked whether re-expression of CD14 depends on the stimulus used to activate the cells. Besides R848, triggering through TLR7/8, we also tested LPS(*E. coli*), triggering predominantly via TLR4, monophosphoryl lipid A (MPLA), a less toxic derivative of LPS, used as an adjuvant in vaccines, Poly(I:C), a TLR3 stimulus as well as a maturation cocktail based on IL-1ß, TNFα, and PgE2. The intrinsic capacity of these stimuli, to induce CD14 expression without exogenous IL-10, varied considerably, with LPS inducing a significant fraction of CD14^+^ cells, whereas cytokine activated cells showing the most significant CD83^+^ fraction and only a few CD14^+^ cells. Poly (I:C) alone also did not increase CD14^+^ cell numbers, but expression of CD83 was poor as well. However, once non-committed cells had been pre-incubated with IL-10, a robust increase in CD14^+^ cells was observed regardless of the activation stimulus used. Quantitatively R848 and LPS still had the most significant impact on the CD14^+^ fraction, but re-expression was also observed with Poly (I:C) or cytokines (Figures 2A,B). Next, we wanted to know, whether non-committed cells could be sensitized for full activation when placing them in a more pro-inflammatory environment early on. _GM/IL4_moC were exposed to titrated amounts of TNFα after 48 h of culture. One group was followed by IL10 incubation 24 h later, whereas the other received no exogenous IL10. Subsequently all cells were stimulated with R848 1 h later. Twenty four hours later, cells were analyzed by FACS. As seen in original plots of one examplary experiment [Figure 2C (upper panel)], TNFα greatly reduced CD14 expression in cells activated with R848 only. When exogenous IL-10 was added, preincubation with as little as 0.1 ng/ml TNFα still reduced CD14 expression significantly, whereas the increase in CD83-expression could not be fully restored (Figure 2C, bottom panel). When combining data from 5 experiments with different donors, 0.1 ng/ml TNFα was sufficient to significantly inhibit CD14 upregulation (Figure 2D).

### IL-10 Boosts the CD14^+^ Subgroup With a Distinct Phenotypic Profile

**Figure 3:**
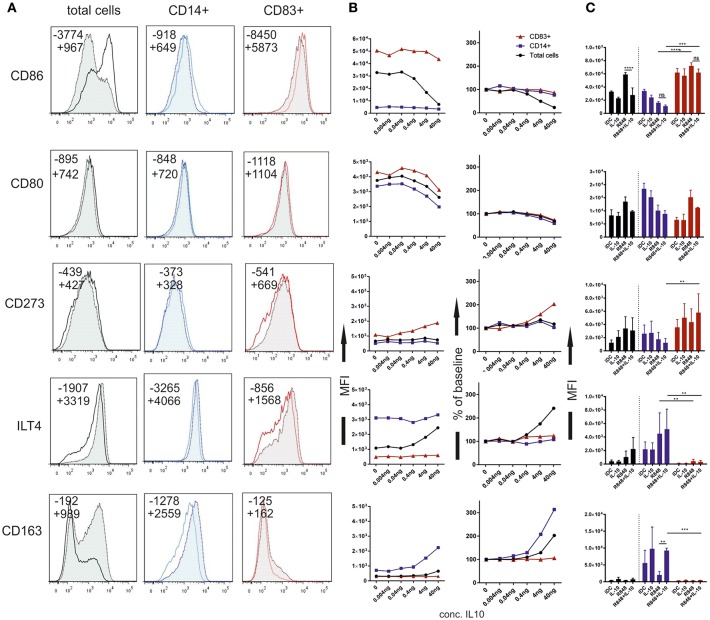
Phenotypic changes of moC depending on IL-10 pre-treatment. moC were pre-treated with IL-10 (40 ng/ml) or not. Cells were then activated using R848 and stained 16 h later. **(A)** Representative histograms for individual markers. Solid lines represent cell populations from the control group without IL-10. Dotted line/tinted filling represent cell populations from the IL-10 treated group. The first panel represents analysis of total cells according to the live scatter gate. The middle panel shows histograms from cells within the CD14^+^ gate. The right panels show histograms from the CD83^+^ population. Numbers in each plot indicate the Median fluorescence intensity; —indicates the control group without IL-10, + indicates the IL-10 group. **(B)** Median fluorescence of individual markers (indicated on the left of the figure), in relation to the IL-10 concentration. Black circles indicate analysis of total cells, red triangles indicate CD83^+^ cells and blue squares indicate CD14^+^ cells. The left panels show mean absolute values from 3 independent experiments. The right panels show the change from the respective baseline (0 ng/ml) in percent. **(C)** Mean with SD from 5 independent experiments using 40 ng/ml IL-10. Black columns (left) represent total cells, blue columns (middle) represent the CD14^+^ population, red columns (right) represent CD83+ cells. (*p < 0.05; **p < 0.01; ***p < 0.001; ****p < 0.0001).

In all experiments so far, CD14 expression and CD83 expression was mutually exclusive, suggesting that CD14 is a reliable marker for an alternative activation pathway of _GM/IL4_moC. By gating on these two populations, we were able to compare fully activated moC^CD83+^ to the alternatively activated moC^CD14+^. A third group, which is CD83^−^/CD14^−^ was not taken into account for this analysis. What became evident immediately, is that moC^CD14+^ exhibit many of the phenotypical features formerly attributed to “maturation-resistant DCs,” “tolerogenic DCs” or CD14^+^MDSCs. Just like these cell populations, moC^CD14+^ displayed lower levels of costimulatory molecules such as CD80 and CD86. But the important finding here is, that within each subgroup of cells, IL-10 had little direct effect on CD80, or CD86 expression (Figure 3). This seemingly contradicts previously published data, as downregulation of costimulatory molecules is often attributed to IL-10 (2, 17, 21, 22). Figure 3A depicts an example of how phenotypes might be analyzed when looking at total cells vs. CD14^+^ and CD83^+^ subgroups. To better evaluate IL10 dose dependency on specific markers, Figure 3B shows titration curves (mean of 3 different experiment and donors), depicting patterns where IL-10 affects expression of a particular marker in all groups (e.g., CD80), predominantly in one group (e.g., CD163, CD273) or where the effects are only seen on the total (mixed) population (e.g., CD86, ILT4), suggesting a quantitative shift in the population rather than a direct effect on expression.

**Figure 4:**
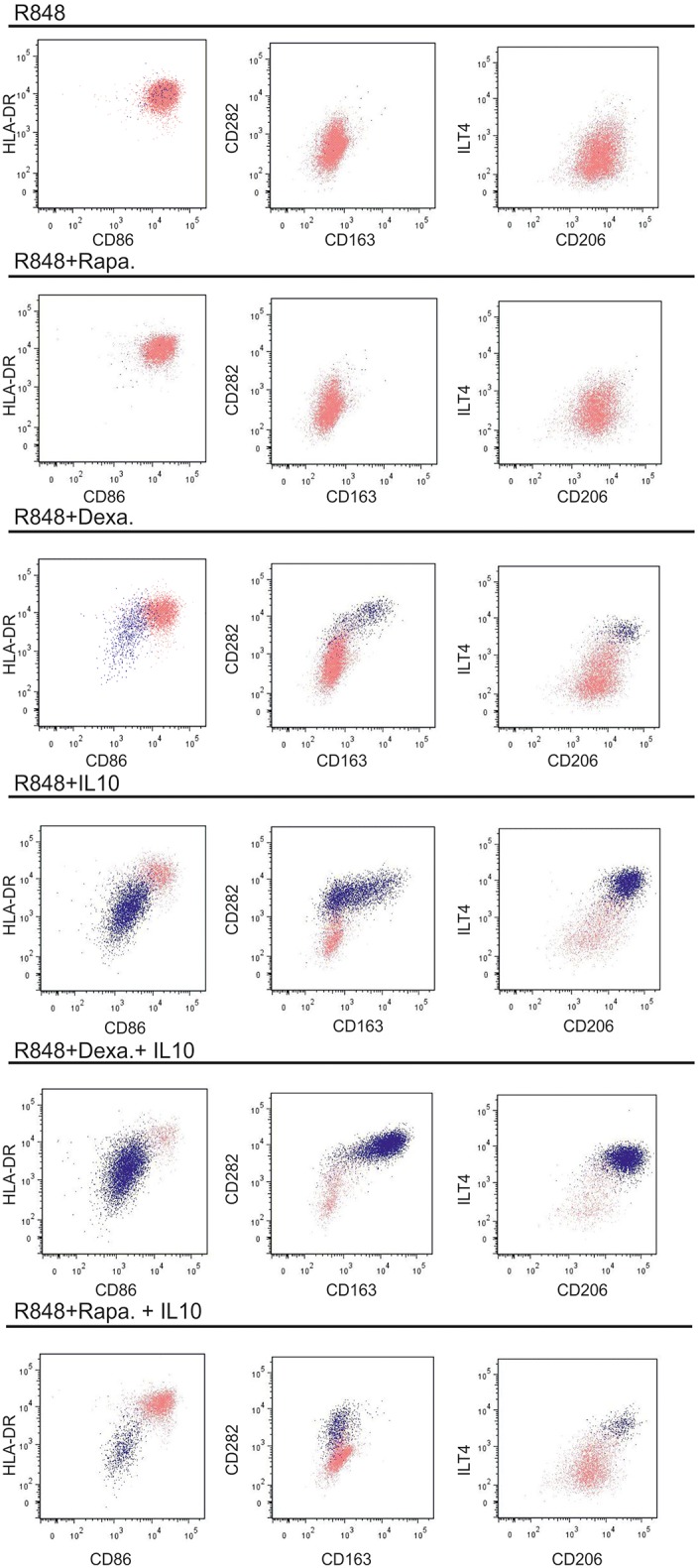
Phenotypic changes in response to other protocols used for generating regulatory DCs. moC were incubated either with IL10 (40 ng/ml), rapamycin (100 ng/ml) or dexamethasone (100 nM) (for 16 h) or left alone. All groups were then activated with R848 (and a second addition of the modulating substance) and stained 24 h later. The light pink population represents CD83^+^ cells, the dark blue population represents CD14^+^ cells. CD14^−^CD83^−^ non-committed cells were excluded in this analysis. Examplary plots of three experiments are shown.

This was statistically analyzed for different treatment groups (at a fixed IL10 dose) (Figure 3C). As has been noted by many groups, the difference between CD86 expression in IL10-treated, activated cells vs. activated cells without IL10 treatment was highly statistically significant when analyzing total cells. However, no difference in the expression level can be observed when looking at CD14 and CD83 subgroups separately. Thus, this difference is explained by the generally lower CD86 expression in moC^CD14+^ and the percentage-wise increase of this cell fraction upon IL-10 preincubation. A very similar pattern was observed for HLA-DR as a marker for MHC class II expression (Figure 4). CD273 (PD-L2) was expressed at a higher level in moC^CD83+^ (Figure 3), whereas no difference was observed for CD274 (PD-L1) (not shown). ILT4 was expressed at a much higher level on moC^CD14+^. In that case, analysis of total cells would suggest a direct role of IL10 to induce higher levels of ILT4, as has been described previously (16). However, again, the difference is mainly explained by the striking difference between moC^CD14+ILT4+^ cells vs. the moC^CD83+ILT4low^ cells. Broadening the spectrum of phenotypic markers, we also included markers described to characterize macrophage differentiation. The scavenger receptor CD163 was exclusively expressed on moC^CD14+^. Exogenous IL-10 enhanced its expression, confirming an IL10-dependent dose-dependency for this receptor (23). CD206(mannose-receptor) remains expressed in _IL10/R848_moC^CD14+^ as is expression of CD282 (TLR2) (Figure 4). CX3CR1, another macrophage-related marker, also was detected exclusively on moC^CD14+^ but was not influenced by IL-10 directly (not shown).

In summary, the phenotypic analysis showed that IL-10 pre-incubation before R848 stimulation gives rise to a macrophage-like, CD14^+^ cell population with increased CD163, CD206, CD282, CX3CR1, and ILT4 expression, and a different level of costimulatory molecules. Contrasting previous interpretations, significant direct effects on marker expression caused by exogenous IL-10, were only seen for CD163, whereas the majority of the effects stems from the shift toward the CD14^+^ population supported by IL-10.

### Other Non-IL-10 Based Approaches for Regulatory DC Induce a Different Phenotype in moC

These phenotypic changes in response to exogenous IL-10, have been typically described for regulatory DC, but cells are often evaluated as one population, not taking differential CD14 expression into account. Using IL-10 to generate such “regulatory DC” is a crucial concept for the use of such immunomodulatory cells clinically (24). In light of the re-expression of CD14 in cultures treated with IL-10, we wanted to assess alternative protocols to generate regulatory DC. We tested two different protocols: pre-incubation either with rapamycin (25) or corticosteroids (dexamethasone) (26–28), each time followed by R848-activation (Figure 4). Rapamycin preceding R848 did not induce any CD14 re-expression, whereas as small CD14^+^ population was observed following dexamethasone pre-incubation and subsequent activation with R848. Of note, when adding IL-10 on top of either dexamethasone or rapamycin, diverging populations were observed: dexamethasone had additive and similar effects on the phenotype of the CD14^+^ cells, thus enhancing expression of CD14 itself, but also CD163, CD282, CD206, and ILT4. In contrast rapamycin suppressed CD14 re-expression to some extent and blocked IL-10 mediated CD163 expression. Thus, phenotypical differences in the type of regulatory cells obtained by the various protocols, are divers, with IL-10 dominating the differentiation toward macrophages.

### IL-10 Affects Function by Shaping Regulatory Subgroups Rather Than Affecting Fully Differentiated Cells Individually

Functionally _IL10/act_moC resemble so-called “regulatory dendritic cells.” Production of inflammatory cytokines ceases, and cells have been shown to inhibit an allogeneic mixed leucocyte reaction and induce T-cell tolerance (15). As these regulatory characteristics have already been described extensively, we wanted to explore functional characteristics with a focus on CD14 expression.

**Figure 5:**
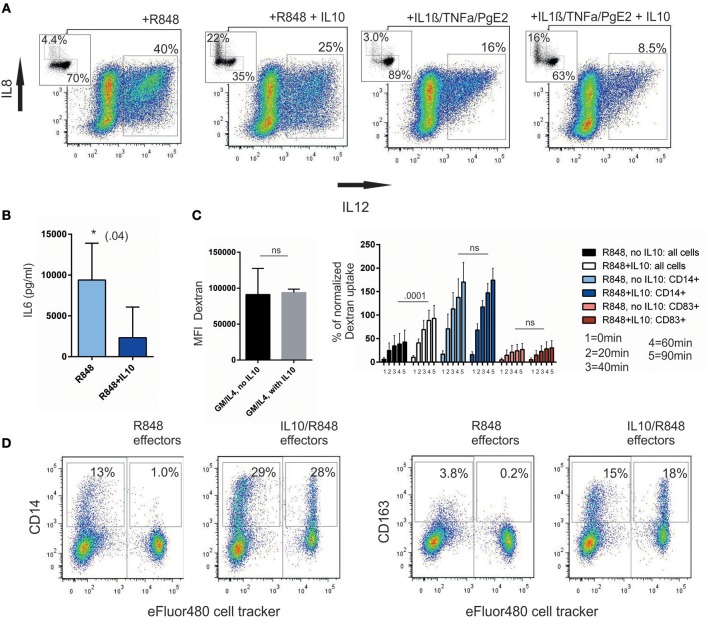
IL-10-mediated effects on the function of moC. **(A)** IL-12 production is reduced. moC were preincubated with IL-10 (or not) and activated either with R848 or a cytokine cocktail. 1 h later, brefeldin A was added for 4 h and cells subsequently stained for intracellular IL-12 and IL-8. Distinction between CD14 and CD83 in the same samples is not possible, as CD14 upregulation is hindered by brefeldin A. CD14(y-axis)- and CD83(x-axis)-staining of a corresponding parallel sample (without brefeldin A) is shown as an inserted dot plot. (*n* = 3) **(B)** IL-6 concentration in the supernatant of differentially treated and activated moC, —pooled data from 5 experiments (*p < 0.05) **(C)**. APC-dextran uptake over time in different cell populations. The left panel shows the MFI for Dextran after 1 h of _GM/IL4_moC with or without IL-10 (7 experiments). The right panel shows the analysis of activated cells with or without IL-10. Analysis was either done on total cells, or gated on CD14^+^ or CD83^+^ cells, respectively. 1–5 indicates the duration of dextran incubation (1 = 0 min, 2 = 20 min, 3 = 40 min, 4 = 60 min, 5 = 90 min). Each value is normalized to the MFI of _GM/IL4_moC at 1 h within the individual experiment (7 experiments). **(D)** Transmission of the CD14^+^ phenotype onto non-committed bystander moC. Two ‘effector' populations were generated either by using LPS/IFNγ as a full type 1- stimulus or IL-10 followed by R848 to induce a CD14^+^ population. After 16 h they were stained with cell tracker dye and mixed at a 1:1 ratio with non-committed, autologous _GM/IL4_moC. Twenty four hours later R848 was added to this co-culture. Cells were then analyzed the next day and separated on the basis of the membrane dye. Examplary plot of 1 out of 3 experiments.

We first re-evaluated production of IL-12 as the critical inflammatory cytokine to drive TH1-responses. It is known, that after IL-10 pre-incubation, IL-12 production is hindered. IL-10 indeed reduces IL-12 production once cells are stimulated, but this inhibition depends on the stimulus used (Figure 5A). Technically, it was not possible, to analyze _IL10/R848_moC^CD14+^ and _IL10/R848_moC^CD83+^ separately, because CD14 upregulation is blocked by the addition of brefeldin A, which is required for the intracellular cytokine staining. However, one likely interpretation is that the shift toward CD14^+^ cells, which do not majorly contribute to IL-12 production (29), explains reduced pro-inflammatory activity. Similarly, total IL-6 production was reduced in the IL-10-pretreated group as assessed by ELISA (Figure 5B). It is well-described that IL-10 treated _GM/IL4_moC start to produce IL-10 endogenously; thus these particular experiments were not repeated.

We next asked how endocytosis, a hallmark of macrophage function, is affected within the different subgroups. Early work by Allavena et al. already showed how the net amount of Dextran-uptake by endocytosis, is increased following IL-10 treatment (11). Subgroup analysis based on CD14 and CD83 expression now allows a refined interpretation: in _GM/IL4_moC cells endocytosis is highest and it is unaffected by exogenous IL-10 (Figure 5C, left panel). In comparison, focusing solely on R848-induced CD14^+^ cells, uptake was lower. Using the MFI of unactivated _GM/IL4_moC after 1 h of Dextran-uptake as the internal reference for the individual experiments, we analyzed how Dextran-uptake varies in R848-activated moC and the influence of IL10: Figure 5C, right panel, first shows pooled data of 7 independent experiments, comparing Dextran uptake within the total cell population. In this analysis, IL10 treatment resulted in a highly significant increase in Dextran uptake, when using the 1 h time point as point of comparison. This could be interpreted as a direct effect of IL-10 on the capacity to do endocytosis, as has been mentioned in previous reports (11, 30). However, Figure 5C also shows that there is no difference between IL10-treated vs. un-treated groups, once CD14+ and CD83+ subgroups are analyzed separately. This means, that there is no dose-dependent effect of exogenous IL-10 on the cells, once the cells have switched to a macrophage-like cell type. This switch is the key event that defines function and phenotype and this step is supported by exogenous IL-10.

Given the heterogenous populations arising from monocytes, we were interested, whether we could detect interactions between these cell populations. Specifically, we asked how _IL10/R848_moC^CD14+^ affect autologous _GM/IL4_moC in the absence of exogenous IL-10. For this experiment, two “effector” populations were generated either by using LPS/IFNγ as a full type 1- stimulus or IL-10 followed by R848 to induce a CD14^+^ population. Twenty four hour after treatment, these two cell preparations were washed and stained with a membrane dye. Subsequently, cells were added at a 1:1 ratio to autologous _GM/IL4_moC^dye−^ for another 24h. Then R848 was added to the groups to induce differentiation in _GM/IL4_moC^dye−^ followed by FACS analysis 24 h later. Based on the staining with the cell tracker, the “effector” population was separated from the _GM/IL4_moC^dye−^ population. In the exemplary experiment shown in Figure 5D, R848 alone induced 13% of CD14^+^ cells in the presence of _LPS/IFN_moC^dye+D14−^ (Figure 5D). In contrast, when _IL10/R848_moC^dye+^ were used as “effectors,” the fraction of CD14^+^ cells within the moC^dye−^ population more than doubled. Similar results were observed when evaluating CD163 expression in the same context. Due to the complexity of this experimental setup, using different preparations of primary cells analyzing sequential events, the overall variation within the three experiments performed is too high, to demonstrate statistical significance. However, the experiment shown in Figure 5D is representative of the effects observed. We conclude that even in conditions, where no exogenous IL-10 is present, activated, CD14-polarized cells are capable of affecting unpolarized bystander cells within a culture period of 48 h.

### Antigen-Specific T-Cells Are Affected During Priming and Expansion by _IL10/R848_moC^D14+^

Next, we wanted to assess the role of _IL10/R848_moC^CD14+^ in the context of antigen-specific T-cell activation. There is ample evidence, how regulatory DC affect-cell activation in the context of mixed leucocyte reactions or in response to a CD3/CD28 stimulus (15). Thus, without repeating these assays, it is a safe assumption that cells generated in our hands would have a similar functional profile. We wanted to extend the findings by looking at a more specific and physiological way to activate T-cells. We focused on antigen-specific priming of naïve CD8^+^ T-cells, using a well-validated experimental system (19, 20). This experimental set-up is calibrated in a way that naïve T-cells specific for the melanosomal peptide antigen Melan-A_(26−35(A27L)_can be efficiently activated, starting from an estimated precursor frequency of 1–10 in 10,000, meaning 20–200 specific T-cells per well-within the starting population, and expanding to a robust, specific cell population of at least 20% at day 10 of culture.

**Figure 6:**
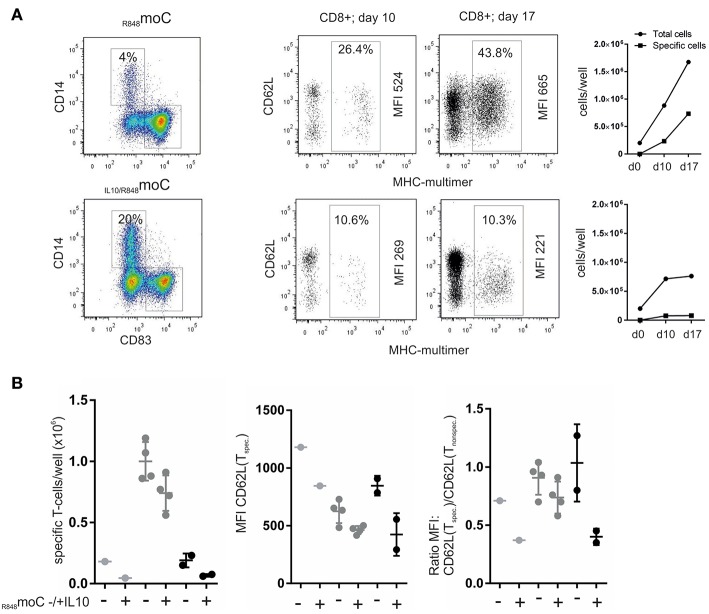
Effects of IL-10-treated moC on antigen-specific priming of naive T-cells. **(A)** moC were differentially treated (left panel) and pulsed with Melan-A peptide. They were then used to prime naive CD8^+^ T-cells. After 10 days of culture using IL-21, IL-7, and IL-15, MHC-multimer^+^ cells were determined and phenotypically characterized. T-cells were then restimulated with peptide-pulsed moC and MHC-multimer^+^ cells were re-evaluated 7 days later. Right panels depict the cell expansion in absolute numbers. **(B)** Summary of three different experiments from different donors. Depending on the cell numbers available (number of APC and number of naïve T-cells), experiments were set up in 1–4 parallel wells. The left panel shows the absolute numbers of antigen-specific (MHC-multimer+) cells per well after 10 days of expansion (based on the precursor frequency the starting cell number in each well varies between 20 and 200 cells). The middle panel summarizes MFI-values for CD62L of the resulting MHC-multimer^+^ cells. The right panel shows the ratio of the CD62L MFI of specific vs. non-specific CD8+ cells within the same sample.

We compared the stimulatory capacity of peptide-pulsed _R848_moC vs. _IL10/R848_moC. As seen for an exemplary experiment in Figure 6A, Expansion of MHC-multimer^+^ T-cells by day 10 was much lower (10.6%) when _IL10/R848_moC were used (which consisted of 20% CD14^+^ cells). In comparison _R848_moC (with a fraction of 4% CD14+ cells) gave yield to 26.4% of antigen-specific T-cells by day 10. Phenotypically, _IL10/R848_moC-expanded T-cells expressed less CD62L in comparison to the _R848_moC primed T-cells. Non-specific bystander T-cells in both groups predominantly retained CD62L expression, indicating that the reduced CD62L expression is due to the specific cell-cell interaction and not due a globally altered microenvironment. The antigen-specific T-cells also proliferated less upon restimulation, which indicates antigen-specific tolerance. The limitations of this assay certainly is the inter-donor variation, as variation in the moC preparation (as documented in Figure 1) combines with donor dependent variation due to the low frequency of antigen-specific naïve CD8^+^ T-cells. However, three separate experiments, (summarized in Figure 6B), analyzing between 1 and 4 separate wells, depending on the available cell material, show a comparable pattern with reduced antigen-specific cell numbers, once IL10 was involved as well as reduced CD62L expression. Therefore, similar to the findings with non-specific clonal stimulation or stimulation of memory T-cells shown in earlier work (15), antigen-specific T-cell priming from the naïve T-cell repertoire is quantitatively and qualitatively affected by IL-10 induced moC^CD14+^ as well.

## Discussion

We here provide a new view of how IL-10 affects monocytic cells in culture: instead of assessing its effects on the bulk culture, the identification of mutually exclusive expression of CD14 or CD83 defines heterogeneity within the culture of monocytic cells, which is greatly augmented by exogenous IL-10 in combination with TLR-triggering. Thus, exogenous IL-10 drives a population shift toward macrophages, but it does not—for the most part—affect individual marker expression (such as CD86) of function (such as endocytosis) within the respective subgroup: _IL10/act_moC^CD83+^ (dendritic cells) do not express less CD86 than their non-IL-10-treated counterparts; _IL10/act_moC^CD14+^ (macrophages) do not take up more Dextran than non-IL10-treated _act_moC^CD14+^.

The strong effects seen in the analyses of total cells, which is repeatedly reported in various papers (2, 17, 21, 22), now finds an explanation as a quantitative shift of different cell populations and not a qualitative change of one homogenous dendritic cell population. The shift toward macrophage-like cells alone, and not differences in expression level on differently treated cells, explains for example, why ILT4, an important myeloid-specific receptor to suppress pro-inflammatory responses (31), suddenly seems increased in the total cell population upon IL10-treatment. Thus, at least three populations need to be distinguished, and analyzed separately, based on CD14 and CD83 expression: committed moC^CD14^, moC^CD83^ and non-committed moC^CD14−CD83−^.

The phenotype- and function-altering effects of IL-10 on immature DC have been known for a long time (15). Early studies by Allavena et al. show, how IL-10 shifted monocytic cells toward macrophages with maintained CD14 expression (11). Of note, in that work, the IL-10 effect was lost, if IL-10 was added at a later time point (e.g., day 3) and no upregulation of CD14 was observed. In a recent paper, _GM/IL4_moC cultured in the presence of IL-10, from the beginning of culture, were termed “MDSC-like.” In this work, upregulation of CD14 has been noted, and interpreted as an indicator for the retention of the monocytic phenotype (12). The protocol to generate prototypic tolerogenic dendritic cells is also based on IL-10, added later at the time of cell activation (15, 24). In an effort for harmonization of the clinical use of tolerogenic dendritic cells, CD11b+CD14++CD163++CD80+CD86+HLA-DR++ cells have been termed DC-10, arising from monocytes in a process termed “arrested immaturity” (8).

The other critical finding in this work is that CD14, rather than serving as a lineage marker (32), can be re-expressed to indicate alternative cell differentiation toward macrophages. Once moC receive a double stimulus consisting of IL-10 first and TLR-agonist second, CD14 and CD83 serve as mutually exclusive markers to define two cell populations with a different phenotypical and functional profile. Thus, CD14 expression may be less a question of halted differentiation (maintained CD14 expression) (27), rather than a sign of active re-expression as part of the differentiation pathway toward a macrophage-like cell.

Monocyte-derived DC are clinically evaluated as therapeutic DC vaccination for cancer (33). For this purpose, CD83, CD86, and HLA-DR often serves as read-out to assess optimal stimuli (17, 34). Our findings are highly relevant in this context, as the precise definition of a potentially suppressive CD83^−^ subgroup within the DC preparation may help to better understand its effects (or lack thereof). Many studies focus on finding the optimized stimulation cocktail, providing the best Th1-oriented stimulation for these cells. Our pre-incubation experiments with TNFα show that rather than the right combination and dose of the stimulus, the timing, and sequence of activation may be most relevant to counteract intrinsic priming by endogenous IL-10 (Figure 2). Once CD14 is fully re-expressed, cells do not convert back to a CD83^+^ inflammatory phenotype.

Likewise in studies on “regulatory DC” the main phenotypical description of such cells is that they express less stimulatory markers (8, 24). Few inhibitory molecules such as ILT4 and CD273 (PD-L2) (17) are known to be expressed at a higher level, but these molecules are not exclusive for a regulatory phenotype. In comparative studies on protocols on the generation of regulatory DC relevant for clinical use, no attention is paid to CD14 expression (24, 35, 36). However, as we now show, CD14, combined with a panel of macrophage markers (Figure 4), positively identifies cells with a stable phenotype and regulatory function.

Functionally, we aimed to add to the known characteristics of IL-10-treated cells. Distinction based on CD14 expression reveals that exogenous IL-10 itself does not directly enhance endocytosis as suggested earlier (11, 37), but changes the cellular composition. Moreover, moC^CD14+^ have the capacity to affect non-committed bystander cells, steering them toward the CD14^+^ phenotype once an additional TLR trigger is provided. Although seen in an artificial experimental system with a broad range of variation, this effect may have implications in tumor biology. Once tumor-associated factors dominate the micromilieu and reverse some of the surrounding myelomonocytic cells to tumor-associated macrophages (TAMs), these TAMs may be able to recruit non-committed bystander cells, especially if an additional TLR-trigger is provided, thereby multiplying the tumor-associated effects. Mere activation of the immune infiltrate, e.g., by a TLR-trigger, may cause unintended, suppressive effects, if cells are primed by IL-10, requiring a more orchestrated intervention (38, 39).

Suppression of T-cell responses is a known hallmark of regulatory DCs. We chose to evaluate functional differences in the context of antigen-specific priming of human, naïve CD8+ T-cells (19, 20). Peptide-pulsed _IL10/R848_moC are poor stimulators of a *de novo* T-cell response and the T-cells are tolerant to a second stimulus. Interestingly, despite reduced proliferation, CD62L expression is lower than in fully activated T-cells. This corroborates, on the level of a *de novo* antigen-specific, human immune response, data on the effects of myeloid-derived suppressor cells from murine models (40, 41).

The caveat of these experiments is, that, to some extent, monocyte-derived DCs, are in itself a culture artifact (42). For murine bone marrow cultures, Helft et al. showed, that these cultures are not monomorphic but comprise of conventional DCs and monocyte-derived macrophages (43). Our data extend these findings to human mononuclear cells showing that culture with GM-CSF and IL-4 is not sufficient to definitively tilt monocytes toward DC differentiation. However, understanding how such cell populations, serving as cell therapeutics, may develop and how they might deviate from the projected path is essential to understand their potential clinical impact. For cancer patients significant difficulties have been described to generate fully activated DCs for clinical use and this deficiency has been linked to the presence of regulatory CD14^+^HLA-DR^lo/neg^ cells (44). For example, an insufficiently activating vaccine may not be a “null” event, but might even have a negative effect (45). Filipazzi et al. described the occurrence of CD14^+^HLA-DR^−/low^ cells with immunosuppressive characteristics following vaccination with GM-CSF and hsp gp96 in melanoma patients (46). Llopiz et al. also observed that IL10-producing “immunosuppressive DC” are induced by therapeutic vaccination with imiquimod-based vaccines, significantly affecting T-cell responses in a murine model (47). Other groups observed elevated levels of IL-10 following vaccination with imiquimod, suggesting that besides the inflammatory activity, a self-regulatory, IL-10-dependent pathway is being triggered. The authors discuss the possibility of using IL-10 blockade clinically, to enhance vaccine effects (48). In this context and in light of our data, it will be interesting to test *in vivo*, whether priming of the vaccination site with TNFα prior to local administration of the TLR-trigger may overcome the described IL-10-dependent pathway.

In summary mutually exclusive, CD14 and CD83-expression in _GM/IL4_moC provides a means to understand functional differences of therapeutically used cell products better. For IL-10, these differences are, for the most part, based on shifts in the magnitude of the respective cell populations rather than a direct regulation on a single molecule level. These findings will help to better design and define cellular products and might help to understand the variable outcome of vaccination in different individuals. Given the prominent role of IL-10 in tumor immunology and the emerging role of CD14^+^HLA-DR^low^MDSC in human diseases, these findings may also have a biologically significant counterpart.

## Data Availability

All datasets generated for this study are included in the manuscript and/or the supplementary files. In very few instances data are briefly mentioned and indicated as “not shown.” This data is available on request.

## Author Contributions

SK, MC, CB, and MW: designed, performed, and analyzed experiments. VW, ME, and PS: analyzed data and provided valuable input throughout the experimental phase. SK and MW: wrote the manuscript. All authors read and approved the manuscript.

### Conflict of Interest Statement

The authors declare that the research was conducted in the absence of any commercial or financial relationships that could be construed as a potential conflict of interest.
